# Stochastic and Regulatory Role of Chromatin Silencing in Genomic Response to Environmental Changes

**DOI:** 10.1371/journal.pone.0003002

**Published:** 2008-08-20

**Authors:** Jung Kyoon Choi, Sohyun Hwang, Young-Joon Kim

**Affiliations:** 1 Department of Biochemistry, Yonsei University, Seoul, Republic of Korea; 2 Korea Research Institute of Bioscience and Biotechnology, Daejeon, Korea; Tel Aviv University, Israel

## Abstract

Phenotypic diversity and fidelity can be balanced by controlling stochastic molecular mechanisms. Epigenetic silencing is one that has a critical role in stress response. Here we show that in yeast, incomplete silencing increases stochastic noise in gene expression, probably owing to unstable chromatin structure. Telomere position effect is suggested as one mechanism. Expression diversity in a population achieved in this way may render a subset of cells to readily respond to various acute stresses. By contrast, strong silencing tends to suppress noisy expression of genes, in particular those involved in life cycle control. In this regime, chromatin may act as a noise filter for precisely regulated responses to environmental signals that induce huge phenotypic changes such as a cell fate transition. These results propose modulation of chromatin stability as an important determinant of environmental adaptation and cellular differentiation.

## Introduction

Stochastic switching of phenotype generates diversity in a genetically clonal population [1]. Population diversity is critical in adaptation to fluctuating environments, especially in regard to phenotypes associated with stress resistance [2], [3]. Stochastic noise or cell-cell variation in gene expression is a key element in phenotypic switching and diversity. A recent study showed how stochastic fluctuations in gene expression can determine cell fate by regulating phenotypic transitions [4]. Heterogeneity of stress resistance was linked to varying expression of stress genes [5]. Increased expression diversity was shown to enable rapid response of a subset of cells to acute stress [6] and found to enhance fitness in the face of fluctuating environments [7].

Phenotypic switching can be dictated by epigenetic switching of gene expression. In *Candida albicans*, deletion of the homolog of *Saccharomyces cerevisiae* Sir2 remarkably increases the frequency of phenotypic switching [8]. The authors propose a model based on the role of the Sir2 protein in telomere position effect, whereby genes in the vicinity of telomeric heterochromatin can switch back and forth between on and off states as a result of unstable silencing [9]–[13]. The model suggests that the relevant genes are located in regions of silent chromatin; thus reduced silencing activity resulting from Sir2 disruption increases switching frequencies of their expression by destabilizing silent chromatin, mimicking telomere position effect in *S. cerevisiae* [8].

Here we sought to explore the genomewide relation of chromatin silencing and stochastic switching of gene expression in *S. cerevisiae*. Genes in low silencing activity regions may have high switching frequencies, contrasting with those in stable silent chromatin. The frequency of switching will eventually be reflected in gene expression noise, which is measured on a genomic scale by a recent study [14]. Increasing evidence highlights the importance of silencing modulation in developing stress-resistant phenotypes via transcription regulation [15]–[17]. Therefore, control of stochasticity in chromatin silencing may play a key part in environmental adaptation of clonal populations.

## Results and Discussion

**Figure 1:**
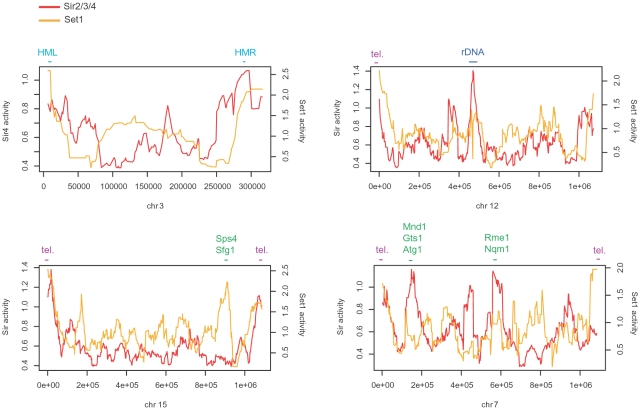
Chromosomal distribution of silencing activity. The red line indicates the average Sir2/3/4 activity of genes in a 50 kb sliding window, which is plotted on the left-side y-axis; likewise, the orange line indicates the average Set1 activity of genes in the same window and its y-axis is on the right-side. The activity of Sir4 was used for a clear pattern for chromosome III (the upper left). The location of the HML, HMR, rDNA loci, and some telomeres (tel.) is denoted above the corresponding peak. Some of peaks in other genomic regions contained two or more consecutively located genes that are involved in control of mating, meiosis, and sporulation. The names of the genes are presented above the plot and their functional description is given in Table S5.

The activity of silencing was estimated based on deletion effects of the Sir complex components (Sir2, Sir3, and Sir4) and Set1 (see Methods). As well as the Sir complex, Set1 is known to be required for HML, HMR, telomere, and rDNA silencing [18], [19]. The genomic distribution of silencing activity, as determined by calculating the average of genes in 50kb genomic regions, confirmed high silencing activity at the HML, HMR, and rDNA loci (Fig. 1). Also, telomeres usually had strong silencing, some examples of which are shown in Fig. 1. We also found many peaks in other genomic regions, indicating genomewide effects of silencing mechanisms.

We compared our silencing measures with transcription rate, chromatin repression level, and histone methylation signals. First, high silencing activity was coupled with low transcription rate (Table S1). This is a result of repression by closed chromatin structure; silencing activity positively correlated with chromatin repression level (Table S1). Chromatin repression is usually associated with histone modifications. In particular, the hypomethylation of H3-K4 and H3-K79 is the characteristics of silent chromatin [11], [20]. The methylation signals showed significant negative correlations with silencing strength (Table S1).

**Figure 2:**
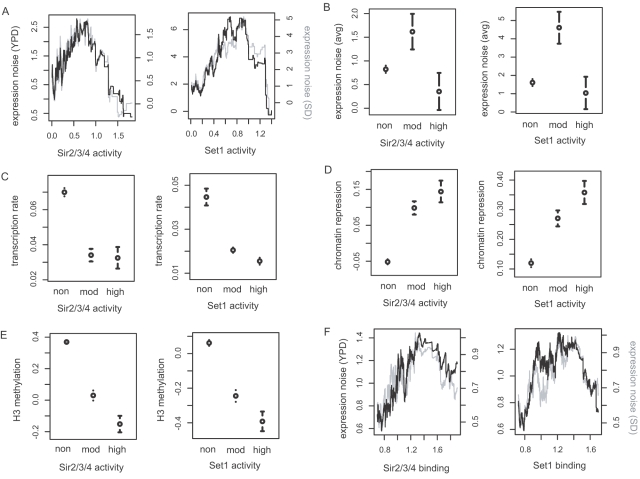
Effects of silencing strength on stochastic noise in gene expression. The strength of silencing activity for each gene was estimated by expression change of the gene according to the deletion of Sir2/3/4 and Set1 (A–E) or by their binding affinity to the gene (F). Expression noise was measured in rich (YPD) and minimal (SD) media (A, F) and the average of the two measures was calculated (avg) (B). The density lines were obtained by averaging noise strength within a sliding window over genes ordered by silencing activity; the right side y-axis of the plot is for the gray line (A, F). The mean plots were obtained for non-, moderately-, and highly-silenced genes (denoted as non, mod, and high); the mean and standard error for each group are shown (B–E).

Given the reliable measures of silencing activity, we now explored its relation with expression noise. Supporting our prediction, we observed a distinctive pattern in the relationship (Fig. 2A): expression noise reaches the peak at intermediate levels of silencing activity and then drops as silencing activity approaches the highest levels. This pattern was so unique as to be found with only four of 263 regulatory proteins. Notably, two of them were known silencing regulators, namely Sir1 and the Sir-recruiting factor Rap1 (Figure S1). On the basis of the pattern (Fig. 2A), we identified non-, moderately-, and highly-silenced genes and compared their average noise strength (Fig. 2B). Low transcription activity of the moderately silenced genes (Fig. 2C–E) suggests repression in many, if not all, cells of the population. The binding signals of the Sir complex and Set1 from ChIP-chip experiments [21] displayed the same patterns: intermediate binding affinity increased expression noise, whereas strong bindings were associated with low expression noise (Fig. 2F).

We compared silencing with gene-specific repression, which is exemplified by the Tup1-Ssn6 (Cyc8) complex. Gene-specific repression targets only one specific promoter by interacting with DNA-binding proteins, whereas silencing involves spreading of silencing marks along the chromatin fiber resulting in repression of multiple genes (reviewed in [20]). We showed that the silencing factors exert consistent effects on multiple adjacent genes within a chromosomal domain, unlike Tup1 and Ssn6 (Table S2). Notably, Tup1 and Ssn6 activity was simply proportional to noise strength (Figure S2). The binding signals of Tup1and its interacting chromatin regulators produced similar patterns (Figure S3).

In general, high noise is found among lowly expressed proteins [14], [22], [23]. A promoter that undergoes infrequent activation tends to produce noisy expression [23]. This can explain the high noise of genes repressed by Tup1-Ssn6 but not the low noise of highly silenced genes. It is also known that the presence of a TATA box increases noise from the promoter [6], [24]. Indeed, repressed genes tend to contain a TATA-box and express high noise (Figure S4). In contrast, silenced genes have low noise even though they tend to have a TATA-box (Figure S4). Promoter-mediated noise may be permitted only outside of heterochromatin. Meanwhile, the proportion of TATA promoters among the moderately silenced genes (25% for Sir2/3/4, 34% for Set1) was not considerably higher than the genomewide average (20%). Moreover, we did not find any transcription factors that express high noise in moderately silenced regions. Thus, promoter-mediated noise seems irrelevant of expression noise associated with weak silencing.

**Figure 3:**
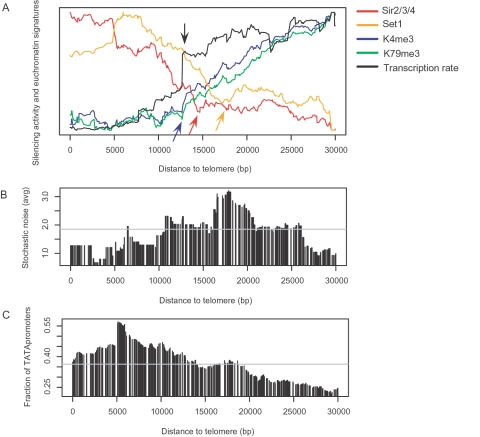
Telomere position effect and stochastic noise in gene expression. For each gene, its distance to the telomere was obtained from the *Saccharomyces* genome database. The average values were calculated within a sliding window of 5 kb over genes ordered by their distance to the telomere. (A) Silencing activity was estimated based on deletion effects of Sir2/3/4 and Set1. The trimethylation of H3-K4 and H3-K79 and transcription rate represent chromatin states. An increase in transcription rate (black arrow), the beginning of an increase in histone methylation signals (blue arrow), and the end of a decrease in Sir activity (red arrow) and Set1 activity (orange arrow) are indicated. (B) The average of the noise measures in rich and minimal media was used. (C) The fraction of TATA-containing promoters was obtained in the same 5 kb window.

Telomere position effect may give rise to expression noise in a promoter-independent manner. We sought to relate the telomeric position of a gene to the degree of noise in its expression. We found that a high degree of noise was displayed approximately between 10 kb∼25 kb from telomeres (Fig. 3B). Intriguingly, this region lies at the interface of heterochromatin and euchromatin. In Fig. 3A, one can notice a sharp increase in transcription rate (black arrow), the beginning of an increase in the histone methylation signals (blue arrow), and the end of a decrease in Sir activity (red arrow) and Set1 activity (orange arrow). They are all indicative of telomeric heterochromatin boundaries. By comparison, changes in Tup1 and Ssn6 activity were not predictive of heterochromatin boundaries (Figure S5). Again, it seems that a high proportion of TATA promoters (∼55%) cannot involve high expression noise in silent chromatin (Fig. 3C).

Increased expression noise from epigenetic instability may not be restricted to telomeric regions. About 80% of the moderately silenced genes were found >50 kb from telomeres. The odds of finding telomere-proximal genes in this group were only slightly higher than in the whole genome (the odds ratio was 1.641). About 46% of the moderately silenced genes showed high expression noise (>1 as defined in [14]). However, they were not enriched near telomeres as well (the odds ratio was 1.317). Although telomere position effect suggests one possible mechanism, expression noise coupled with incomplete silencing could occur throughout the genome, presumably by different mechanisms.

Now we turned to examine the functional implications of chromatin silencing. First, we calculated the average silencing activity of genes in each Gene Ontology category. Functional categories associated with Sir2/3/4 and Set1 activity are summarized in Table S3 and S4, respectively. A significant overlap was found between the two lists: approximately 50% of categories in one list appeared in the other list, implying functional similarity between the Sir complex and Set1. Especially, functions related to control of sporulation, meiosis, and reproduction were among commonly found categories. We indeed found some genomic regions of high silencing activity containing two or more consecutive genes that are involved in such processes (Fig. 1). Functional description of these genes is given in Table S5.

High Sir2/3/4 activity was mostly found with functions related to life cycle control, but relatively lower activity was associated with response to external stimuli or stress (see Table S3). We also observed categories related to signal transduction and DNA repair. On the other hand, Set1 activity showed preferential enrichment for metabolic processes and metabolite transport (Table S4). Except for life cycle control, these functions markedly overlap with annotation of a cluster of genes that are commonly induced across a variety of stress conditions [25]. Activation of silent genes may be involved in the common molecular mechanism of stress response via diverse biological processes. The reported general stress-response genes [25] showed a certain level of silencing (P value = 1.9×10^−5^ for Sir2/3/4 and P value = 0.01 for Set1) and a remarkably high degree of expression noise (P value = 1.1×10^−30^). On one hand, this underscores the importance of expression diversification promoted by moderate silencing in stress response. On the other hand, this raises a question regarding the role of strongly silenced genes with homogenous expression patterns.

To address this question, we characterized individual transcriptional responses to specific stresses from the stress expression profiles [25]. To define gene sets responsive to a specific stress, we identified genes that show a significant expression change in each condition. Additionally, a cohort of genes bound by a transcription factor under a specific environmental condition [26] also served as a stress-responsive gene set. The silencing activity and cell-cell variability of genes in each of the 200 gene sets are given as –log_10_ (P value) (Table S6). Our approach was to compare the magnitude of silencing and cell-cell variability across the defined stress-responsive gene sets.

**Figure 4:**
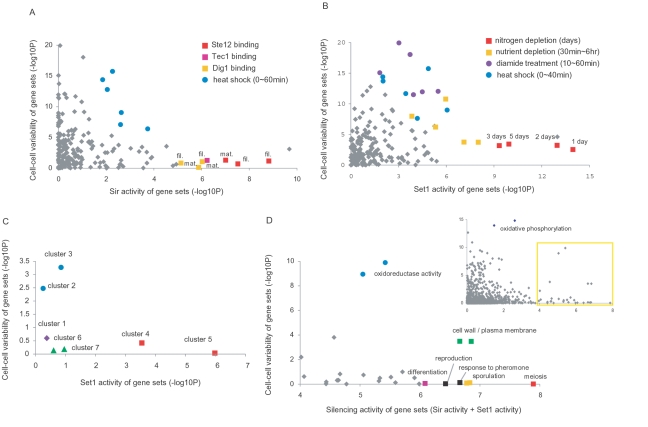
Effects of silencing strength on stress response. The silencing activity and expression noise of genes in a defined group were compared with the rest of genes and its statistical significance was reported as –log_10_ (P value). (A–B) Analysis for the 200 stress-responsive gene sets (Table S6). The threshold was 5.301 (α = 0.001). (A) Plot of expression noise as a function of Sir-complex activity. Gene sets shown as rectangles contain the target genes of Ste12, Tec1, and Dig1 under the condition of filamentation inducing (fil.) or mating inducing (mat.). (B) Plot of expression noise as a function of Set1 activity. (C) Analysis of sporulation gene clusters. Cluster 1 corresponds to genes induced at early time points and cluster 7 at late time points. Cluster 4 contains known key genes required for pre-meiotic processes and cluster 5 contains meiosis-specific factors required for proper sporulation. The threshold was 3.85 (α = 0.001). (D) Analysis for Gene Ontology categories. The sum of the Sir2/3/4 score and Set1 score was used on the x-axis. Among a total of 1157 categories (inset), those for which combined silencing activity is greater than 4 (circumscribed in yellow) are enlarged (listed in Table S7). The threshold was 6.063 (α = 0.001).

The overall pattern shown in Fig. 4 is that gene sets highly regulated by silencing factors maintain a low degree of expression noise, recapitulating the patterns shown in Fig. 2A–B. Genes that are bound by Ste12, Tec1, and Dig1 when the cell is stimulated for filamentation or mating turned out to be under strong influence of the Sir complex (Fig. 4A). This is consistent with high ranking categories in Table S3. On the other hand, genes that are strongly regulated by Set1 were responsive to nitrogen depletion (Fig. 4B), which is an environmental cue that induces filamentation or sporulation. This pattern was not clear for shorter periods (<6 hours) of nitrogen or amino acid starvation (the red versus orange rectangles). By using the time course microarray analysis of sporulation [27], we confirmed the same patterns for long-term starvation and commitment to sporulation. Clusters 4 and 5, containing early- and middle-meiotic genes that are induced at the time of commitment [27], exhibit high Set1 activity and low cell-cell variation (Fig. 4C).

Despite the seemingly similar roles of Sir2/3/4 and Set1 in control of reproduction and growth, we observe that high Sir2/3/4 activity is mainly involved in regulation of the mating process through signal transduction (see the top ranking categories in Table S3), contrasting with metabolic roles of Set1. Some of metabolic functions highly suppressed by Set1 may be involved in nitrogen utilization under sporulation-inducing conditions. For example, the expression of genes in the allantoin pathway (see the top ranking categories in Table S4) is sensitively induced by lack of nitrogen, which allows yeast cells to use allantoin as a sole nitrogen source [28].

We next identified sets of genes whose expression is heterogeneous and is moderately regulated by silencing factors (circles in Fig. 4A–B). They were found to be highly responsive to acute heat shocks and the sulfhydryl oxidizing agent diamide. Diamide was shown to elicit expression response resembling a composite of responses to heat shock, oxidative stress, and disulfide reducing agent, demonstrating pleiotropic effects [25]. This is in line with relatively lower ranks of stress-response categories in the Sir2/3/4 activity table (Table S3) and high expression noise of the common stress-response genes. In Fig. 4C, cluster 3 displays the highest cell-cell variation among the sporulation clusters. This cluster, induced earlier than the time of commitment, was found to contain known genes involved in starvation and stress responses [27].

The same analysis was carried out for Gene Ontology categories (inset of Fig. 4D). The categories where the sum of Sir2/3/4 and Set1 scores is greater than 4 are enlarged in Fig. 4D (listed in Table S7). The pattern of strong silencing and low noise was found for categories such as meiosis, sporulation, response to pheromone, reproduction, and cell differentiation. These developmental changes essentially require remodeling of the cell wall, which is also a mechanism of increasing stress resistance of the cell [16]. The pattern found for cell wall genes (Fig. 4D) highlights the influence of silencing modulation on their regulation during stress response.

Meanwhile, the pattern of moderate silencing and high noise was found for genes with oxidoreductase activity. From the speculation that this group of genes may be involved in response to oxidative stress, we compared responsiveness of these genes across the various stress conditions (Table S8). As expected, we observed high responsiveness of the genes to hydrogen peroxide and the superoxide-generating drug menadione. We also found enrichment of genes regulated by Mal33, Pho2, and Rds1under highly hyperoxic conditions. Additionally, diamide treatment and short-term amino acid starvation were also found in the list.

The general picture emerging from these findings is that i) genes with high cell-cell variability in unstable silent chromatin are responsive to acute environmental changes and ii) genes whose expression is homogeneously maintained in stable silent chromatin respond to a prolonged or intensive stress that requires dramatic phenotypic changes such as cell fate transitions. Cautious cellular decision-making will be needed before a transition to another form of growth or reproduction. Thus, the relevant genes should be precisely regulated by signaling processes showing deferred response, in contrast to the swift and flexible response of stochastically expressed genes. This may explain the association of high silencing activity with signal-transduction proteins and transcription factors. It is surprising to find that silent chromatin can act as both a noise generator and a noise filter, controlling phenotypic diversity and fidelity in the direction of conferring an adaptive advantage to a cell population. It is tempting to postulate the existence of an epigenetic filter for noise control during cell differentiation in multicellular organisms [29], implicating a role for the Polycomb silencers that are involved in position effect variegation [11] and cell fate control [30]. Our results offer a new perspective on a stochastic and regulatory role of chromatin structure modulation in environmental adaptation and cellular differentiation.

## Methods

Detailed information on Methods is described in Text S1.

### Estimation and evaluation of Sir2/3/4- and Set1-mediated silencing activity

Expression change of each yeast gene accompanying the deletion of Sir2/3/4 and Set1 was measured[31], [32]. The average of Sir2, Sir3, and Sir4 was used for the effect of the Sir complex. For evaluation, we obtained transcription rate from previous data [33], [34], chromatin repression level from mutant expression profiles for H3 and H4[35], and the trimethylation of H3-K4 and H3-K79 from ChIP-chip experiments[36].

### Classification of genes based on silencing activity

We observed that for both Sir2/3/4 and Set1, genes with 0.5<silencing activity<1.0 showed highest levels of expression noise (Fig. 2A). Thus, we defined non-silenced genes as silencing activity<0.5, moderately silenced genes as 0.5<silencing activity<1.0, and highly silenced genes as silencing activity>1.0.

### Functional implications of silencing activity in terms of Gene Ontology categories

Gene Ontology categories were downloaded from the *Saccharomyces* genome database. Using the Gene Ontology hierarchy, we mapped each gene to all its parent categories. We calculated the average silencing activity of Sir2/3/4 and Set1 for genes in each category. Considering the distribution of functional characteristics over the ordered list, we selected categories with the average >0.5.

### Silencing activity and expression noise for stress-responsive gene sets or Gene Ontology categories

See Text S1 for defining gene sets. For each set, we carried out the Wilcox rank sum test and Kolmogorov-Smirnov test between the genes in the set and the rest of genes. The significance of the test was reported as –log_10_ (P value). A higher –log_10_ (P value) indicates that the genes in the set have higher silencing activity or expression noise compared with other genes. The Bonferroni correction was used to set the threshold to 0.001.

## Supporting Information

Text S1 Supplementary Methods
(0.08 MB PDF)

Figure S1 The activity of transcription factors and chromatin regulators for a gene was estimated based on the gene's expression change in each null mutant (Hu et al.). Expression noise was measured in rich (YPD) and minimal (SD) media (Newman et al.). The density lines were obtained by averaging expression noise within a sliding window over genes ordered by the strength of regulatory activity. The right side y-axis corresponds to the gray line. The additional Rap1 plot (the leftmost) is from the study of Wyrick et al.
(0.52 MB PDF)

Figure S2 Gene-specific repression level for a gene was measured based on the gene's expression change by the deletion of Tup1 or Ssn6 (Hughes et al.). Expression noise was measured in rich (YPD) and minimal (SD) media. The density lines were obtained by averaging expression noise within a sliding window over genes ordered by the degree of Tup1 or Ssn6 activity. The right side y-axis corresponds to the gray line.
(0.08 MB PDF)

Figure S3 Expression noise as a function of binding signals of chromatin modifiers related to gene-specific repression. Tup1-binding affinity was measured by ChIP-chip experiments (Buck et al.). The Tup1-Ssn6 complex interacts with Hda, Rpd3, and Isw2. Their binding affinity was from a ChIP-chip data collection (Tsankov et al.). Expression noise was measured in rich (YPD) and minimal (SD) media. The density lines were obtained by averaging noise strength within a sliding window over genes ordered by binding affinity. The right side y-axis corresponds to the gray line.
(0.21 MB PDF)

Figure S4 Comparison of gene-specific repression and chromatin silencing in terms of the relationship between TATA-promoter presence and expression noise. Silencing (or gene-specific repression) activity for a gene was measured based on the gene's expression change accompanying the deletion of Sir2/3/4 and Set1 (or Tup1 and Ssn6). The average of the noise measures from rich (YPD) and minimal (SD) media was used. The presence of a TATA box was identified by a previous study and the fraction of TATA-containing promoters was obtained in a sliding window over genes ordered by the strength of silencing or repression. The right side y-axis corresponds to the gray line.
(0.41 MB PDF)

Figure S5 Comparison of gene-specific repression and chromatin silencing in terms of telomere position effect. For each gene, its distance to the telomere was obtained from the Saccharomyces genome database (http://www.yeastgenome.org). Silencing (or gene-specific repression) activity for a gene was measured as the gene's expression change following the loss of Sir2/3/4 and Set1 (or Tup1 and Ssn6). The average signals were calculated within a sliding window of 5kb over genes ordered by their distance to the telomere.
(0.22 MB PDF)

Table S1 Correlation of silencing activity measures and other silencing indices.
(0.04 MB PDF)

Table S2 Number of silent or repressed domains for a sliding window of varying size.
(0.01 MB PDF)

Table S3 Functional implications of Sir2/3/4 silencing activity. The average of genes belonging to each Gene Ontology category was calculated. Shown is the ordered list of selected categories (avg. Sir2/3/4>0.5). The categories were classified into five groups and color-coded as summarized at the top of the table. The v marks on the right side of the values indicate that the relevant category was also found in the Set1 activity table (Table S4).
(0.01 MB PDF)

Table S4 Functional implications of Set1 silencing activity. The average of genes belonging to each Gene Ontology category was calculated. Shown is the ordered list of selected categories (avg. Set1>0.5). The categories were classified into five groups and color-coded as summarized at the top of the table. The v marks on the right side of the values indicate that the relevant category was also found in the Sir2/3/4 activity table (Table S3).
(0.01 MB PDF)

Table S5 Functional description of consecutively located genes in genomic regions where high silencing activity measures of Sir2/3/4 or Set1 are found (see Fig. 1).
(0.01 MB PDF)

Table S6 Analysis of stress-responsive gene sets. Genes in each set were compared with the rest of genes and its significance was reported as -log10 (P value). The table contains stress conditions as defined from the expression profiles (Gasch et al.) and transcription-factor location analyses (Harbison et al.), the silencing activity of genes in each set (Sir2/3/4 and Set1), the noise of genes in each set as measured in rich medium (Noise (ypd)), and the number of genes in each set (# responsive genes).
(0.01 MB PDF)

Table S7 Analysis of Gene Ontology categories. Genes in each category were compared with the rest of genes and its significance was reported as -log10 (P value). The table contains Gene Ontology categories, the silencing activity of genes in each category (Sir2/3/4 and Set1), the sum of the two silencing scores (Silencing), and the noise of genes in each set as measured in rich medium (Noise (ypd)). Selected categories are shown in the same color-code as the rectangles and circles in Fig. 4D.
(0.01 MB PDF)

Table S8 Stress response of oxidoreductase genes. We analyzed genes belonging to the two categories identified as ‘oxidoreductase activity’ in Table S7. The table reports stress conditions as defined from the expression profiles and transcription-factor location analyses, and the responsiveness of the genes to each stress condition, which is represented as -log10 (P value). Shown in red are stress conditions where the responsiveness score is greater than the threshold.
(0.02 MB PDF)
