# Supplementary material for: Stochastic and Regulatory Role of Chromatin Silencing in Genomic Response to Environmental Changes
Source: PLoS One. 2008 Aug 20;3(8):e3002. doi: 10.1371/journal.pone.0003002 (PMC2500160; doi:10.1371/journal.pone.0003002)
Supplement: Text S1 — Supplementary Methods (0.08 MB PDF) [file pone.0003002.s001.pdf]

# TEXT S1. Supplementary Methods

**Estimation and evaluation of Sir2/3/4- and Set1-mediated silencing activity.** Expression change of each yeast gene accompanying the deletion of Sir2/3/4 and Set1 was measured[1,2]. The global expression profile for Set1-null mutants[2] showed that a majority of genes were derepressed by Set1 deletion (e.g., 220 induced and 4 repressed genes at two-fold changes). In contrast, Sir2/3/4 deletion gives rise to up- and down-regulation (e.g., Sir2 deletion yielded 303 induced and 361 repressed genes at two-fold changes). The repressed genes also showed strong correlations with low transcription rate, high chromatin repression, and hypomethylation of H3-K4 and H3-K79, exhibiting symmetric patterns with the induced genes (data not shown). The loss of the Sir proteins may lead to enhanced silencing at particular loci due to their silencing-inhibitory function or via certain indirect effects. For example, Sir4 inhibits rDNA silencing function of Sir2; thus, Sir4 deletion strengthens silencing at the rDNA loci[3]. Therefore, we decided to use the absolute value of expression change by Sir2/3/4 deletion and the magnitude of up-regulation by Set1 deletion. The average of Sir2, Sir3, and Sir4 was used for the effect of the Sir complex. To check whether our silencing measures truly reflect the level of silencing, we used data for transcription rate, chromatin repression level, and histone methylation. Transcription rate for each gene was measured using mRNA abundance and mRNA half-life[4], as suggested by Fraser et al.[5]. Chromatin repression level for each gene can be measured as its derepression by loss of histone proteins. The average of mutant expression profiles for H3 and H4[6] was used. The trimethylation of H3-K4 and H3-K79 was measured by ChIP-chip experiments[7]. The average of the two binding signals was used. We checked to see if our silencing measures are negatively correlated with transcription rate and histone methylation, and positively correlated with chromatin repression level (Table S1).

**Pattern comparisons with genomewide deletions of regulatory proteins.** We screened expression profiles of yeast knockout strains of 263 regulatory proteins, including transcription factors and chromatin

modifiers[8], for the relation of their activity with expression noise. The activity of each regulator for each gene was estimated by expression change of the gene resulting from disruption of the regulator, using the absolute (or raw) value in the same manner as Sir2/3/4 (or Set1). For each of the 263 regulators, we examined how the noise pattern changes according to the strength of regulatory activity. Similar patterns with Sir2/3/4 and Set1 were found for Rap1, Sir1, Pdr1, and Ppr1 (Figure S1). Rap1 and Sir1 are known silencing regulators.

**Classification of genes on the basis of silencing activity.** According to the pattern of stochastic noise as a function of silencing activity, we defined three sets of genes: non-, moderately-, and highly-silenced genes. We observed that for both Sir2/3/4 and Set1, genes with 0.5 < silencing activity < 1.0 showed highest levels of expression noise (Fig. 2A). Thus, we defined non-silenced genes as silencing activity < 0.5, moderately silenced genes as 0.5 < silencing activity < 1.0, and highly silenced genes as silencing activity > 1.0. For the Sir complex, each group contained 5073, 701, and 310 genes, respectively. For Set1, each group contained 5341, 457, and 195 genes, respectively. The mean and standard error of expression noise, transcription rate, chromatin repression, and histone methylation were computed for each group.

**Identification of chromosomal domains of silent region.** A silent domain can be defined as chromosomal region where silencing activity is consistently high among underlying genes. We find the z score to be appropriate for this purpose since it considers the variance of sample values in the form of the standard error of the mean. The mean and the standard error of silencing activity were calculated for genes within a window of varying size sliding along the yeast chromosomes. Only windows having at least 10 genes were taken into account. The ratio of the mean over the standard error was obtained for each window: z=Mean/(SD/sqrt(n)), where Mean and SD indicate the mean and standard deviation of silencing activity measures and n is the number of genes in the window. Significant domains were identified with $\alpha$=0.001 by the Bonferroni adjustment. The activity of Tup1 and Ssn6 was calculated from

mutant expression profiles[9] in the same manner as Set1. The number of the identified domains for Sir2/3/4, Set1, Tup1, and Ssn6 are reported in Table S2. For the 50kb window, random permutations of the chromosomes yielded on average 6 and 0 domains for Sir2/3/4 and Set1, respectively.

**Transcription factors associated with silencing-mediated noise.** We tested the possibility that promoters for specific transcription factors are related to high expression noise of moderately silenced genes. To this end, we used ChIP-chip data for DNA-binding profiles of yeast transcription factors[10]. For each transcription-factor cohort, we implemented the Wilcox rank sum test to determine whether the cohort genes have higher noise level compared to the rest of genes. Similarly, we tested whether the cohort genes have a moderate level of silencing activity. For this, the Wilcox rank sum test was implemented while leaving out genes with the highest levels of silencing activity (i.e., Sir2/3/4 > 1.0; Set1 > 1.0). The test results were called significant when the Bonferroni-corrected P value < $10^{-3}$. Confirming the results of Newman et al.[11], Hap4, Cin5, and Hap1 were detected for high noise. However, their cohort genes had only a low level of silencing activity: the minimum Bonferroni-corrected P value=0.255 for Hap4 and Sir2/3/4. Therefore, these transcription factors may be able to produce noisy expression only outside of heterochromatin.

**Functional implications of silencing activity in terms of Gene Ontology categories.** Gene Ontology categories were downloaded from the *Saccharomyces* genome database (http://www.yeastgenome.org). Using the Gene Ontology hierarchy, we mapped each gene to all its parent categories. For example, a gene assigned to ‘mitotic cell cycle’ should be also mapped to ‘cell cycle’. The categories with < 5 or >1000 activity measures for Sir2/3/4 and Set1 were excluded from the further analysis. We calculated the average silencing activity of Sir2/3/4 and Set1 for genes in each category. Considering the distribution of functional characteristics over the ordered list, we selected categories with the average > 0.5 (this corresponds to the threshold between non- and moderately-silenced genes). Table S3 and S4 show the

selected categories for Sir2/3/4 and Set1, respectively. Approximately 50% of the categories appeared in both lists (v marks in the tables), implying similar roles of the two factors in silencing related function.

**Silencing activity and expression noise of common stress-response genes.** From 142 expression profiles for various environmental conditions, Gasch et al.[12] identified a cluster of genes that are stereotypically induced across a range of different stresses (dubbed “genes induced in the ESR”). Using the program TreeView (available at http://rana.stanford.edu/software/), we extracted the corresponding cluster from the author’s clustering data (http://genome-www.stanford.edu/yeast_stress/). We compared the silencing activity (Sir2/3/4 and Set1) and expression noise (measured in rich medium) between this cluster and the rest of genes by using the Wilcox rank sum test. The P values are reported in the main text.

**Silencing activity and expression noise for stress-responsive gene sets.** We first defined sets of genes responsive to specific stress conditions. From the expression profiles for diverse stress conditions[12], we identified genes with > four-fold expression change for each condition. Additionally, a cohort of genes bound by a transcription factor in a specific environmental condition[10] also served as a stress-responsive gene set. For each set, we implemented the Wilcox rank sum test between the genes in the set and the rest of genes. The significance of the test was reported as $-\log_{10}$ (P value). A higher $-\log_{10}$ (P value) indicates that the genes in the set have higher silencing activity or expression noise compared with other genes. The test was carried out for sets with at least 5 genes. This criterion yielded the significance scores for 200 sets (Table S6). For a multiple testing of the 200 sets, the Bonferroni correction set the threshold to 5.301 ($\alpha$=0.001). Using the Kolmogorov-Smirnov test produced similar results. Expression noise measured in rich medium was used.

**Silencing activity and expression noise for Gene Ontology categories.** Gene Ontology categories were downloaded and preprocessed as described above. The categories with < 5 noise measures or >1000

silencing-activity measures were excluded from the further analysis. Because of the large size of some categories, the Wilcox rank sum test produced infinite $-\log_{10}$ (P value) in some cases. So we used the Kolmogorov-Smirnov test instead. For a multiple testing of 1157 categories, the Bonferroni adjustment set the threshold to 6.063 ($\alpha$=0.001). The categories where the sum of the Sir2/3/4 and Set1 scores is greater than 4 are listed in Table S7.

**Stress responsiveness of oxidoreductase genes**. The categories, "oxidoreductase activity, acting on CH-OH group of donors" and "oxidoreductase activity, acting on the CH-OH group of donors, NAD or NADP as acceptor", contained 78 genes that were available in the above stress-response datasets[10,12]. The responsiveness of these genes to a specific stress condition was compared to the rest of genes by using the Wilcox rank sum test. The responsiveness was measured as the absolute value of expression changes to the stress condition[12] or the binding status of transcription factors in the stress condition[10]. For a multiple testing of the 259 stress conditions, the threshold was set to 5.413 ($\alpha$=0.001 with the Bonferroni adjustment). The top 40 conditions are listed in Table S8.

## Supplementary References


1. Wyrick JJ, Holstege FC, Jennings EG, Causton HC, Shore D, et al. (1999) Chromosomal landscape of nucleosome-dependent gene expression and silencing in yeast. Nature 402: 418-421.
2. Bernstein BE, Humphrey EL, Erlich RL, Schneider R, Bouman P, et al. (2002) Methylation of histone H3 Lys 4 in coding regions of active genes. Proc Natl Acad Sci USA 99: 8695-8700.
3. Smith JS, Brachmann CB, Pillus L, Boeke JD. (1998) Distribution of a limited Sir2 protein pool regulates the strength of yeast rDNA silencing and is modulated by Sir4p. Genetics 149: 1205-1219.
4. Wang Y, Liu CL, Storey JD, Tibshirani RJ, Herschlag D, et al. (2002) Precision and functional specificity in mRNA decay. Proc Natl Acad Sci USA 99: 5860-5865.
5. Fraser HB, Hirsh AE, Giaever G, Kumn J, Eisen MB. (2004) Noise minimization in eukaryotic gene expression. PLoS Biol 2: e137.
6. Sabet N, Volo S, Yu C, Madigan JP, Morse RH. (2004) Genome-wide analysis of the relationship between transcriptional regulation by Rpd3p and the histone H3 and H4 amino termini in budding yeast. Mol Cell Biol 24: 8823-8833.
7. Pokholok DK, Harbison CT, Levine S, Cole M, Hannett NM, et al. (2005) Genome-wide map of nucleosome acetylation and methylation in yeast. Cell 122: 517-527.
8. Hu Z, Killion PJ, Iyer VR. (2007) Genetic reconstruction of a functional transcriptional regulatory network. Nat Genet 39: 683-687.
9. Hughes TR, Marton MJ, Jones AR, Roberts CJ, Stoughton R, et al. (2000) Functional discovery via a compendium of expression profiles. Cell 102: 109-126.
10. Harbison CT, Gordon DB, Lee TI, Rinaldi NJ, Macisaac KD, et al. (2004) Transcriptional regulatory code of a eukaryotic genome. Nature 431: 99-104.
11. Newman JR, Ghaemmaghami S, Ihmels J, Breslow DK, Noble M, et al. (2006) Single-cell proteomic analysis of S. cerevisiae reveals the architecture of biological noise. Nature 441: 840-846.
12. Gasch AP, Spellman PT, Kao CM, Carmel-Harel O, Eisen MB, et al. (2000) Genomic expression programs in the response of yeast cells to environmental changes. Mol Biol Cell 11: 4241-4257.
