# Supplementary figures and images for: Stochastic and Regulatory Role of Chromatin Silencing in Genomic Response to Environmental Changes
Source: PLoS One. 2008 Aug 20;3(8):e3002. doi: 10.1371/journal.pone.0003002 (PMC2500160; doi:10.1371/journal.pone.0003002)

Figure S1

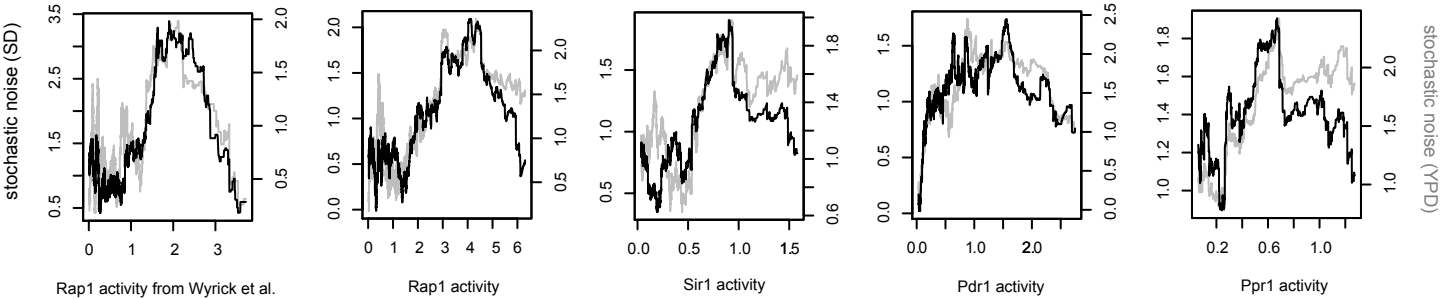

Supplement: Figure S1 — The activity of transcription factors and chromatin regulators for a gene was estimated based on the gene's expression change in each null mutant (Hu et al.). Expression noise was measured in rich (YPD) and minimal (SD) media (Newman et al.). The density lines were obtained by averaging expression noise within a sliding window over genes ordered by the strength of regulatory activity. The right side y-axis corresponds to the gray line. The additional Rap1 plot (the leftmost) is from the study of Wyrick et al. (0.52 MB PDF) [file pone.0003002.s002.pdf]

Figure S2

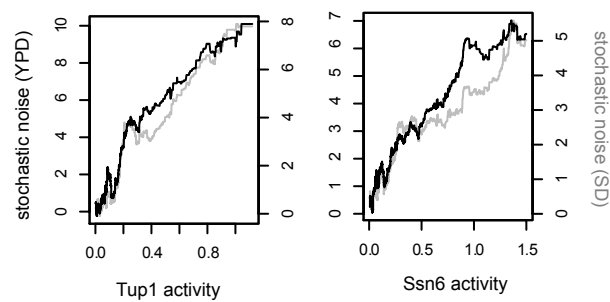

Supplement: Figure S2 — Gene-specific repression level for a gene was measured based on the gene's expression change by the deletion of Tup1 or Ssn6 (Hughes et al.). Expression noise was measured in rich (YPD) and minimal (SD) media. The density lines were obtained by averaging expression noise within a sliding window over genes ordered by the degree of Tup1 or Ssn6 activity. The right side y-axis corresponds to the gray line. (0.08 MB PDF) [file pone.0003002.s003.pdf]

Figure S3

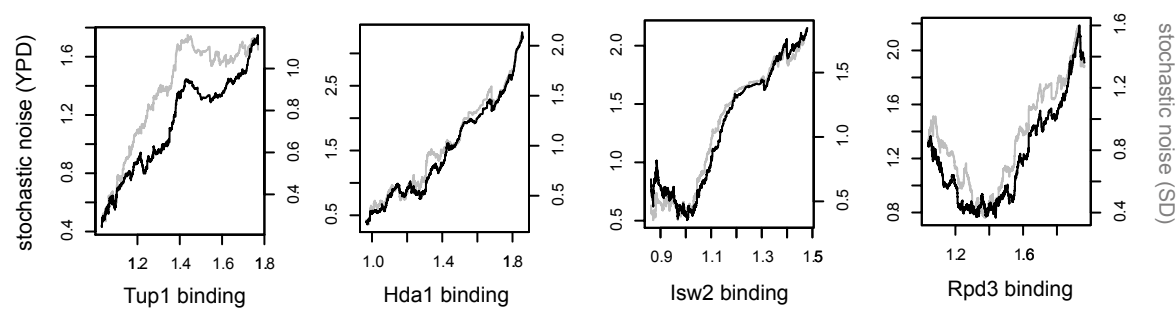

Supplement: Figure S3 — Expression noise as a function of binding signals of chromatin modifiers related to gene-specific repression. Tup1-binding affinity was measured by ChIP-chip experiments (Buck et al.). The Tup1-Ssn6 complex interacts with Hda, Rpd3, and Isw2. Their binding affinity was from a ChIP-chip data collection (Tsankov et al.). Expression noise was measured in rich (YPD) and minimal (SD) media. The density lines were obtained by averaging noise strength within a sliding window over genes ordered by binding affinity. The right side y-axis corresponds to the gray line. (0.21 MB PDF) [file pone.0003002.s004.pdf]

Figure S4

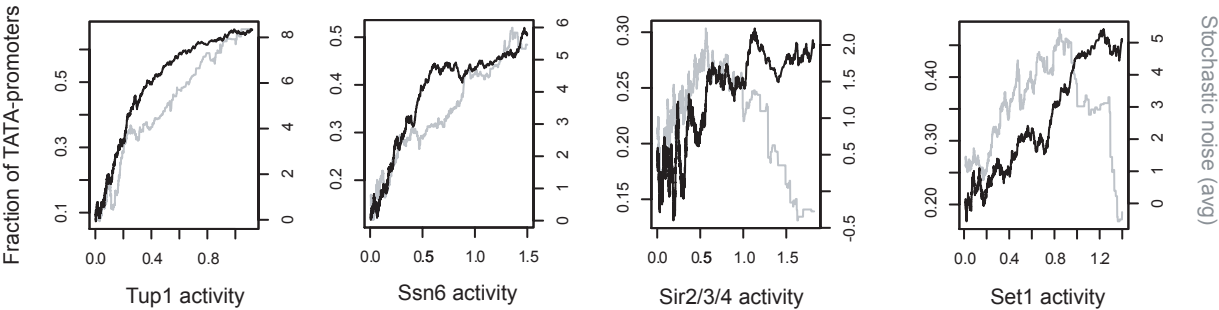

Supplement: Figure S4 — Comparison of gene-specific repression and chromatin silencing in terms of the relationship between TATA-promoter presence and expression noise. Silencing (or gene-specific repression) activity for a gene was measured based on the gene's expression change accompanying the deletion of Sir2/3/4 and Set1 (or Tup1 and Ssn6). The average of the noise measures from rich (YPD) and minimal (SD) media was used. The presence of a TATA box was identified by a previous study and the fraction of TATA-containing promoters was obtained in a sliding window over genes ordered by the strength of silencing or repression. The right side y-axis corresponds to the gray line. (0.41 MB PDF) [file pone.0003002.s005.pdf]

Figure S5

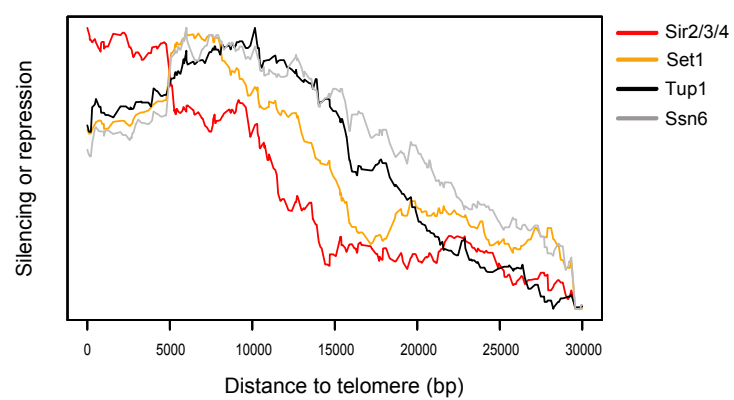

Supplement: Figure S5 — Comparison of gene-specific repression and chromatin silencing in terms of telomere position effect. For each gene, its distance to the telomere was obtained from the Saccharomyces genome database (http://www.yeastgenome.org). Silencing (or gene-specific repression) activity for a gene was measured as the gene's expression change following the loss of Sir2/3/4 and Set1 (or Tup1 and Ssn6). The average signals were calculated within a sliding window of 5kb over genes ordered by their distance to the telomere. (0.22 MB PDF) [file pone.0003002.s006.pdf]
