# Supplementary material for: Stochastic and Regulatory Role of Chromatin Silencing in Genomic Response to Environmental Changes
Source: PLoS One. 2008 Aug 20;3(8):e3002. doi: 10.1371/journal.pone.0003002 (PMC2500160; doi:10.1371/journal.pone.0003002)
Supplement: Table S1 — Correlation of silencing activity measures and other silencing indices. (0.04 MB PDF) [file pone.0003002.s007.pdf]

Table S1. Correlation of silencing activity measures and other silencing indices.

**Silencing activity**	**transcription rate**	**chromatin repression**	**histone methylation**
Sir2/3/4	r = −0.362<br>P = 3.3×10^{-151}	r = 0.242<br>P = 1.2×10^{-79}	r = −0.356<br>P = 1.3×10^{-159}
Set1	r = −0.384<br>P = 3.7×10^{-169}	r = 0.336<br>P = 4.6×10^{-155}	r = −0.483<br>P = 4.0×10^{-308}

Spearman's rank correlation (r) and its P value are reported.
