# Supplementary material for: Stochastic and Regulatory Role of Chromatin Silencing in Genomic Response to Environmental Changes
Source: PLoS One. 2008 Aug 20;3(8):e3002. doi: 10.1371/journal.pone.0003002 (PMC2500160; doi:10.1371/journal.pone.0003002)
Supplement: Table S2 — Number of silent or repressed domains for a sliding window of varying size. (0.01 MB PDF) [file pone.0003002.s008.pdf]

Table S2. Number of silent or repressed domains for a sliding window of varying size

Regulator	10kb	20kb	30kb	40kb	**50kb**	100kb
Sir2/3/4	0	49	67	42	**66**	200
Set1	0	9	34	42	**49**	14
Tup1	0	0	4	0	**0**	0
Ssn6	0	0	1	1	**2**	0
