# Supplementary material for: Stochastic and Regulatory Role of Chromatin Silencing in Genomic Response to Environmental Changes
Source: PLoS One. 2008 Aug 20;3(8):e3002. doi: 10.1371/journal.pone.0003002 (PMC2500160; doi:10.1371/journal.pone.0003002)
Supplement: Table S3 — Functional implications of Sir2/3/4 silencing activity. The average of genes belonging to each Gene Ontology category was calculated. Shown is the ordered list of selected categories (avg. Sir2/3/4>0.5). The categories were classified into five groups and color-coded as summarized at the top of the table. The v marks on the right side of the values indicate that the relevant category was also found in the Set1 activity table (Table S4). (0.01 MB PDF) [file pone.0003002.s009.pdf]

Supplementary Table 3

mating, sporulation, filamentous growth, meiosis, reproduction, differentiation
signal transduction (during conjugation, receptor linked), DNA repair
response to stress and stimulus, detoxification of reactive oxygen species
metabolic, catabolic, biosynthetic processes, metabolite transport
maintanence of telomere or condensed chromosome

Gene Ontology category	avg. Sir2/3/4	
regulation of conjugation with cellular fusion	1.53010	
signal transduction during conjugation with cellular fusion	1.53010	
pheromone-dependent signal transduction during conjugation with cellular fusion	1.53010	
regulation of conjugation	1.53010	
G-protein coupled receptor protein signaling pathway	1.30935	v
response to pheromone during conjugation with cellular fusion	1.17459	
vitamin B6 metabolic process	1.03496	v
pyridoxine metabolic process	1.03496	v
response to pheromone	1.02318	
cell surface receptor linked signal transduction	0.98881	v
extracellular region	0.98801	
meiotic DNA double-strand break formation	0.95308	v
DNA catabolic process, endonucleolytic	0.90907	v
mating projection tip	0.89150	
sexual reproduction	0.85914	
conjugation	0.85914	
conjugation with cellular fusion	0.85914	
peroxisomal matrix	0.83553	v
oxidoreductase activity, oxidizing metal ions, NAD or NADP as acceptor	0.82392	v
ferric-chelate reductase activity	0.82392	v
cell projection part	0.81648	
cell projection	0.80118	
mating projection	0.80118	
DNA catabolic process	0.78156	v
multi-organism process	0.77759	
thiamin biosynthetic process	0.73795	v
synapsis	0.73622	v
meiotic recombination	0.71632	
thiamin and derivative biosynthetic process	0.71347	v
thiamin metabolic process	0.71081	v
spore wall assembly (sensu Fungi)	0.70596	v
spore wall assembly	0.70596	v
meiosis I	0.68582	v
meiotic gene conversion	0.68109	v
telomeric DNA binding	0.67857	v
thiamin and derivative metabolic process	0.67684	v
mating type determination	0.66550	
reproductive developmental process	0.66550	
sex determination	0.66550	
invasive growth (sensu Saccharomyces)	0.66305	
pre-autophagosomal structure	0.65584	v
integral to plasma membrane	0.65265	

microtubule binding	0.64240	
**mating type switching**	0.63819	
intrinsic to plasma membrane	0.63248	
**reproduction**	0.63065	v
**meiosis**	0.62981	v
**meiotic cell cycle**	0.62981	v
**M phase of meiotic cell cycle**	0.62981	v
**fructose transmembrane transporter activity**	0.62916	v
**mannose transmembrane transporter activity**	0.62916	v
**gene conversion at mating-type locus**	0.62712	
plasma membrane part	0.62681	
**double-strand break repair via homologous recombination**	0.62353	
**allantoin metabolic process**	0.61981	v
**allantoin catabolic process**	0.61981	v
**heterocycle catabolic process**	0.61981	v
**sugar transmembrane transporter activity**	0.61602	v
**signal transducer activity**	0.61517	
**molecular transducer activity**	0.61517	
**hexose transmembrane transporter activity**	0.61455	v
**monosaccharide transmembrane transporter activity**	0.61455	v
**oxidoreductase activity, oxidizing metal ions**	0.60956	v
**monosaccharide transport**	0.60572	v
**hexose transport**	0.60572	v
DNA-directed DNA polymerase activity	0.60097	
**external encapsulating structure**	0.59973	
**chitin- and beta-glucan-containing cell wall**	0.59973	
**cell wall**	0.59973	
**spore wall**	0.59382	v
**spore wall (sensu Fungi)**	0.59382	v
**solute:cation symporter activity**	0.59236	v
**symporter activity**	0.59236	v
**recombinational repair**	0.58350	
DNA recombination	0.57732	
**peroxisomal part**	0.57709	
**microbody part**	0.57709	
**glucose transmembrane transporter activity**	0.57429	v
**integral to peroxisomal membrane**	0.56602	
**intrinsic to peroxisomal membrane**	0.56602	
**double-strand break repair via single-strand annealing**	0.56598	
**phosphoinositide-mediated signaling**	0.55709	
**inositol lipid-mediated signaling**	0.55709	
**double-stranded DNA binding**	0.55621	
**cell development**	0.55476	
**fatty acid beta-oxidation**	0.55108	
**carbohydrate transport**	0.54848	v
mitotic recombination	0.54654	
**vitamin biosynthetic process**	0.54637	v
**water-soluble vitamin biosynthetic process**	0.54637	v

**double-strand break repair via nonhomologous end joining**	0.54532	
**carbohydrate transmembrane transporter activity**	0.54512	v
**reproductive process**	0.54510	v
**cell communication**	0.54001	
extrinsic to membrane	0.53978	
**nitrogen compound catabolic process**	0.53789	
**amine catabolic process**	0.53789	
**signal transduction**	0.53766	
**condensed chromosome**	0.53646	
**oxidoreductase activity, acting on the aldehyde or oxo group of donors, NAD or NADP as acceptor**	0.53581	
**peroxisome**	0.53481	
**microbody**	0.53481	
anaphase-promoting complex	0.53266	
**nonprotein amino acid metabolic process**	0.53155	
**cellular developmental process**	0.53077	v
**cell differentiation**	0.53077	v
**establishment of organelle localization**	0.52967	
**prospore membrane**	0.52830	v
**immature spore**	0.52830	v
**prospore**	0.52830	v
**nuclear chromosome, telomeric region**	0.52686	
**fermentation**	0.52647	v
**glucosidase activity**	0.52557	v
**cellular response to extracellular stimulus**	0.52504	
**cellular response to starvation**	0.52504	
**response to starvation**	0.52504	
**cellular response to nutrient levels**	0.52504	
**reproductive sporulation**	0.52308	v
**sporulation (sensu Fungi)**	0.52308	v
**non-recombinational repair**	0.52275	
microtubule associated complex	0.52257	
regulation of a molecular function	0.52234	
**sporulation**	0.52229	v
**response to chemical stimulus**	0.52163	
**reproductive process in single-celled organism**	0.52029	v
**reproductive cellular process**	0.52029	v
**condensed nuclear chromosome**	0.51698	v
**M phase**	0.51536	
**regulation of catalytic activity**	0.51398	
**filamentous growth**	0.51356	
**regulation of transcription by carbon catabolites**	0.51318	v
**multidrug transporter activity**	0.51294	
deacetylase activity	0.51230	
**response to external stimulus**	0.51186	
**response to extracellular stimulus**	0.51186	
**response to nutrient levels**	0.51186	
**hydrolase activity, acting on carbon-nitrogen (but not peptide) bonds, in linear amides**	0.50700	
**telomere maintenance via recombination**	0.50532	
**fatty acid oxidation**	0.50512	
endodeoxyribonuclease activity	0.50498	v
**cellular response to stress**	0.50192	
**cellular response to stimulus**	0.50192	
