# Supplementary material for: Stochastic and Regulatory Role of Chromatin Silencing in Genomic Response to Environmental Changes
Source: PLoS One. 2008 Aug 20;3(8):e3002. doi: 10.1371/journal.pone.0003002 (PMC2500160; doi:10.1371/journal.pone.0003002)
Supplement: Table S4 — Functional implications of Set1 silencing activity. The average of genes belonging to each Gene Ontology category was calculated. Shown is the ordered list of selected categories (avg. Set1>0.5). The categories were classified into five groups and color-coded as summarized at the top of the table. The v marks on the right side of the values indicate that the relevant category was also found in the Sir2/3/4 activity table (Table S3). (0.01 MB PDF) [file pone.0003002.s010.pdf]

Table S4

metabolic, catabolic, biosynthetic processes, metabolite transport
sporulation, meiosis, reproduction, differentiation
detoxification of reactive oxygen species, response to stress and stimulus
receptor linked signal transduction
maintanence of telomere or condensed chromosome

Gene Ontology category	avg. Set1	
allantoin metabolic process	1.01550	v
allantoin catabolic process	1.01550	v
heterocycle catabolic process	1.01550	v
solute:cation symporter activity	0.99083	v
symporter activity	0.99083	v
fructose transmembrane transporter activity	0.93482	v
mannose transmembrane transporter activity	0.93482	v
aryl-alcohol dehydrogenase activity	0.93438	
sugar transmembrane transporter activity	0.89429	v
carbohydrate transmembrane transporter activity	0.85789	v
hexose transmembrane transporter activity	0.85085	v
monosaccharide transmembrane transporter activity	0.85085	v
glucose transmembrane transporter activity	0.85085	v
glucosidase activity	0.83744	v
vitamin B6 metabolic process	0.83314	v
pyridoxine metabolic process	0.83314	v
carbohydrate transport	0.81267	v
monosaccharide transport	0.79281	v
hexose transport	0.79281	v
glutathione metabolic process	0.78500	
meiotic DNA double-strand break formation	0.73417	v
cysteine-type peptidase activity	0.71318	
peroxisomal matrix	0.71278	v
siderophore transport	0.70950	
pre-autophagosomal structure	0.70850	v
aldehyde metabolic process	0.69267	
oxidoreductase activity, oxidizing metal ions, NAD or NADP as acceptor	0.69233	v
ferric-chelate reductase activity	0.69233	v
heterocycle metabolic process	0.68933	
response to toxin	0.68543	
prospore membrane	0.68415	v
immature spore	0.68415	v
prospore	0.68415	v
hydrolase activity, hydrolyzing O-glycosyl compounds	0.68000	
iron ion transport	0.67556	
polyamine transmembrane transporter activity	0.67167	
secondary metabolic process	0.66617	
thiamin metabolic process	0.66565	v
DNA catabolic process, endonucleolytic	0.65825	v

**aldo-keto reductase activity**	0.65783	
unfolded protein binding	0.65777	
**spore wall assembly (sensu Fungi)**	0.65677	v
**spore wall assembly**	0.65677	v
**reproductive sporulation**	0.64264	v
**sporulation (sensu Fungi)**	0.64264	v
**thiamin and derivative metabolic process**	0.64017	v
**secondary active transmembrane transporter activity**	0.62729	
**thiamin biosynthetic process**	0.62719	v
**thiamin and derivative biosynthetic process**	0.62719	v
**G-protein coupled receptor protein signaling pathway**	0.62629	v
**cell surface receptor linked signal transduction**	0.62629	v
**response to nutrient**	0.62314	
**oxidoreductase activity, oxidizing metal ions**	0.62263	v
**DNA catabolic process**	0.62189	v
**sporulation**	0.61975	v
**nucleobase metabolic process**	0.61400	
**polyamine transport**	0.61129	
**hydrolase activity, acting on glycosyl bonds**	0.60574	
**de novo pyrimidine base biosynthetic process**	0.60517	
sister chromatid cohesion	0.60267	
**regulation of transcription by carbon catabolites**	0.60117	v
**vitamin transport**	0.59767	
**amine transmembrane transporter activity**	0.59700	
**reproductive process in single-celled organism**	0.59188	v
**reproductive cellular process**	0.59188	v
**monocarboxylic acid transmembrane transporter activity**	0.59175	
**spore wall**	0.59067	v
**spore wall (sensu Fungi)**	0.59067	v
**vitamin biosynthetic process**	0.58707	v
**water-soluble vitamin biosynthetic process**	0.58707	v
**oxidoreductase activity, acting on the CH-OH group of donors, NAD or NADP as accept**	0.58570	
**cellular developmental process**	0.58389	v
**cell differentiation**	0.58389	v
**di-, tri-valent inorganic cation transport**	0.58262	
**oxidoreductase activity, acting on CH-OH group of donors**	0.58129	
synapsis	0.57100	v
**oxidoreductase activity, acting on peroxide as acceptor**	0.57000	
**peroxidase activity**	0.57000	
**reproductive process**	0.56892	v
**vitamin metabolic process**	0.56298	
**water-soluble vitamin metabolic process**	0.56298	
**glycogen metabolic process**	0.56242	
**NADH metabolic process**	0.55783	
**carboxy-lyase activity**	0.55590	
**reproduction of a single-celled organism**	0.55397	
**meiosis**	0.55351	v
**meiotic cell cycle**	0.55351	v
**M phase of meiotic cell cycle**	0.55351	v

**sulfur metabolic process**	0.54413	
**meiotic gene conversion**	0.54367	v
transcription factor binding	0.54250	
**sulfur compound biosynthetic process**	0.54120	
**active transmembrane transporter activity**	0.53997	
**nitrogen utilization**	0.53388	
**pyrimidine base biosynthetic process**	0.53100	
**cellular carbohydrate catabolic process**	0.52968	
**carbohydrate catabolic process**	0.52968	
**condensed nuclear chromosome**	0.52944	v
**transition metal ion transport**	0.52924	
plasma membrane	0.52275	
RNA methyltransferase activity	0.52000	
**carbon-carbon lyase activity**	0.51307	
**fermentation**	0.50950	v
**telomeric DNA binding**	0.50667	v
endodeoxyribonuclease activity	0.50644	v
**meiosis I**	0.50587	v
**transition metal ion transmembrane transporter activity**	0.50308	
**reproduction**	0.50206	v
**cell division**	0.50160	
deoxyribonuclease activity	0.50127	
**energy reserve metabolic process**	0.50080	
**O-acyltransferase activity**	0.50063	
