# Supplementary material for: Stochastic and Regulatory Role of Chromatin Silencing in Genomic Response to Environmental Changes
Source: PLoS One. 2008 Aug 20;3(8):e3002. doi: 10.1371/journal.pone.0003002 (PMC2500160; doi:10.1371/journal.pone.0003002)
Supplement: Table S5 — Functional description of consecutively located genes in genomic regions where high silencing activity measures of Sir2/3/4 or Set1 are found (see Fig. 1). (0.01 MB PDF) [file pone.0003002.s011.pdf]

Table S5. Functional description of consecutively located genes in genomic regions where high silencing activity measures of Sir2/3/4 or Set1 are found (see Fig. 1).

**Gene name**	**Functional description (quoted from the *Saccharomyces* genome database, http://www.yeastgenome.org)**
SPS4	SPorulation Specific transcript; protein whose expression is induced during sporulation
SFG1	SuperFicial pseudohyphal Growth; putative transcription factor required for growth of superficial pseudohyphae
MND1	Meiotic Nuclear Divisions; protein required for recombination and meiotic nuclear division
GTS1	zinc-finger protein that regulates ultradian rhythm, cell size, cell cycle, lifespan, sporulation, heat tolerance, and multidrug transport
ATG1	protein required for authophagy; null mutants are defective in sporulation
RME1	Regulator of MEiosis; zinc-finger protein involved in regulation of mating and meiosis; mediates cell type control of sporulation
NQM1	protein induced by alpha-factor and during diauxic shift; null mutant non-quiescent cells exhibit reduced reproductive capacity
