# Supplementary material for: Stochastic and Regulatory Role of Chromatin Silencing in Genomic Response to Environmental Changes
Source: PLoS One. 2008 Aug 20;3(8):e3002. doi: 10.1371/journal.pone.0003002 (PMC2500160; doi:10.1371/journal.pone.0003002)
Supplement: Table S6 — Analysis of stress-responsive gene sets. Genes in each set were compared with the rest of genes and its significance was reported as -log10 (P value). The table contains stress conditions as defined from the expression profiles (Gasch et al.) and transcription-factor location analyses (Harbison et al.), the silencing activity of genes in each set (Sir2/3/4 and Set1), the noise of genes in each set as measured in rich medium (Noise (ypd)), and the number of genes in each set (# responsive genes). (0.01 MB PDF) [file pone.0003002.s012.pdf]

Table S6

stress condition	Sir2/3/4	Set1	Noise (ypd)	# responsive genes
aa.starv.0.5.h	0.00000	2.98161	1.18851	512
aa.starv.1.h	1.28073	3.83267	7.97212	111
aa.starv.2.h	0.51308	2.15272	4.03289	63
aa.starv.4.h	1.05383	4.67773	5.34344	83
aa.starv.6.h	2.52070	7.10786	3.75957	91
AFT2_H2O2Hi	0.26955	1.50343	0.61792	61
AFT2_H2O2Lo	0.46470	2.23378	0.66326	115
ARG81_SM	0.54657	0.53150	1.48537	28
ARO80_SM	0.77765	0.41461	0.76815	32
ASH1_BUT14	0.61198	0.01550	2.99401	51
BAS1_SM	0.30969	0.23997	0.97205	17
CAD1_SM	0.45041	0.38114	0.12580	26
CBF1_SM	0.22914	0.15725	0.73943	281
CHA4_SM	0.11255	0.13180	0.41099	37
CIN5_H2O2Hi	0.41621	1.63351	1.80358	81
CIN5_H2O2Lo	0.29729	3.33165	0.73403	129
constant.0.32.mM.H2O2..10.min..redo	0.30419	1.46291	4.33038	156
constant.0.32.mM.H2O2..100.min..redo	0.81004	0.19825	3.18034	24
constant.0.32.mM.H2O2..120.min..redo	1.25541	0.04043	4.25121	22
constant.0.32.mM.H2O2..160.min..redo	1.44664	0.41410	3.60009	30
constant.0.32.mM.H2O2..20.min..redo	0.00915	4.58786	3.06701	345
constant.0.32.mM.H2O2..30.min..redo	0.00072	3.82430	5.64979	254
constant.0.32.mM.H2O2..40.min..rescan	0.37382	0.60500	3.33876	160
constant.0.32.mM.H2O2..50.min..redo	0.16995	2.63389	8.82571	74
constant.0.32.mM.H2O2..60.min..redo	0.54486	2.64168	6.14760	63
constant.0.32.mM.H2O2..80.min..redo	0.70365	0.27791	1.84527	23
DAL80_RAPA	1.97896	1.23125	0.42035	40
DAL81_RAPA	0.07479	1.38662	3.52490	96
DAL82_RAPA	0.49979	1.88488	1.46291	56
DAL82_SM	0.42902	0.30051	0.98615	59
diauxic.shift.timecourse.18.5.h	0.01591	0.73513	11.37926	172
diauxic.shift.timecourse.20.5.h	0.00000	0.66779	3.84728	315
DIG1_Alpha	6.02862	0.53441	1.06249	76
DIG1_BUT14	5.97608	0.31313	0.14481	67
DIG1_BUT90	5.13425	0.26775	0.84133	42
dtt.000.min..dtt.2	1.65033	1.08314	1.78923	51
dtt.015.min.dtt.2	3.70074	4.07503	2.60859	65
dtt.030.min..dtt.2	1.73351	1.59622	0.20196	25
dtt.120.min.dtt.2	0.14952	0.24999	1.21557	18
dtt.240.min.dtt.2	0.00006	0.09039	0.19829	133
FHL1_RAPA	0.00000	0.00010	0.00267	191
FHL1_SM	0.00000	0.00077	0.00277	204
FKH2_H2O2Hi	0.57806	0.14475	1.36429	108
FKH2_H2O2Lo	0.42415	0.47552	0.70600	31

GAL4_GAL	0.50723	0.73188	0.01207	41
GAL4_RAFF	1.34679	1.62412	0.16101	39
GAT1_RAPA	0.15297	0.24419	0.69298	27
GAT1_SM	0.47582	1.41275	0.57756	47
GCN4_RAPA	0.03040	0.03515	4.34942	161
GCN4_SM	0.06738	0.09111	5.56044	187
GCR2_SM	0.69782	0.32646	0.53650	56
GLN3_RAPA	0.07484	0.11096	4.17307	67
GLN3_SM	0.02713	1.02865	2.09374	50
GZF3_H2O2Hi	0.30158	0.07712	0.79396	13
GZF3_RAPA	3.70845	1.96119	0.83550	39
HAP2_RAPA	0.97110	0.64191	4.33205	42
HAP4_H2O2Lo	0.05545	0.35541	0.05169	24
HAP5_SM	0.29880	0.42907	3.82253	39
Heat.Shock.000.minutes..hs.2	2.26514	4.86874	15.74488	143
Heat.Shock.000.minutes..hs.2.1	0.71286	3.44953	11.67214	121
Heat.Shock.000.minutes.hs.2	0.25202	1.00552	12.08872	91
Heat.Shock.005.minutes..hs.2	0.76265	0.24017	0.92613	18
Heat.Shock.015.minutes..hs.2	0.00000	4.86734	1.98341	203
Heat.Shock.030inutes..hs.2	0.00001	1.23213	2.43026	60
Heat.Shock.05.minutes.hs.1	0.54407	5.32702	6.20872	174
Heat.Shock.060.minutes..hs.2	0.43892	1.44031	1.64903	60
Heat.Shock.10.minutes.hs.1	0.00001	12.98378	4.59920	582
Heat.Shock.15.minutes.hs.1	0.00000	2.48692	5.03499	752
heat.shock.17.to.37..20.minutes	0.00000	2.17196	7.37643	896
Heat.Shock.20.minutes.hs.1	0.00000	3.15311	6.84277	690
heat.shock.21.to.37..20.minutes	0.00000	0.74424	8.55527	792
heat.shock.25.to.37..20.minutes	0.00000	1.18602	7.05751	793
heat.shock.29.to.37..20.minutes	0.00000	1.96210	8.27610	552
Heat.Shock.30.minutes.hs.1	0.00012	6.05080	8.98114	554
heat.shock.33.to.37..20.minutes	0.02239	2.02243	13.71856	211
Heat.Shock.40.minutes.hs.1	0.11288	4.17893	7.62653	223
Heat.Shock.60.minutes.hs.1	0.06060	0.56079	7.75219	45
Heat.Shock.80.minutes.hs.1	1.25477	0.88717	5.73445	47
HSF1_H2O2Hi	0.00573	0.00519	1.27971	125
HSF1_H2O2Lo	0.00475	0.01463	1.19807	102
Hypo.osmotic.shock...15.min	1.40033	1.23200	4.33665	23
Hypo.osmotic.shock...30.min	3.91581	3.55790	1.78282	66
Hypo.osmotic.shock...45.min	2.04767	1.69873	2.53399	48
Hypo.osmotic.shock...5.min	4.52859	0.43660	3.55265	25
Hypo.osmotic.shock...60.min	0.62291	0.27423	1.17412	23
IME4_H2O2Hi	0.35899	0.58383	2.50773	34
LEU3_SM	0.02088	0.16482	2.28507	27
MAC1_H2O2Hi	0.94190	1.25049	0.60139	18
MAL33_H2O2Hi	0.15753	0.45116	0.07728	38
MBP1_H2O2Hi	0.05338	0.03154	0.05593	135

MBP1_H2O2Lo	0.24309	0.02540	0.70927	61
MCM1_Alpha	1.53670	0.39706	0.88543	107
MET31_SM	0.15863	3.07606	0.27081	14
MET32_SM	0.92204	0.50290	0.91755	41
MET4_SM	0.05220	0.37042	0.13900	28
MOT3_H2O2Hi	0.16361	1.46665	0.78673	12
MSN2_Acid	0.39784	0.00937	1.21907	31
MSN2_H2O2Hi	0.40902	0.38912	5.36353	79
MSN2_H2O2Lo	0.18410	0.00812	3.97973	47
MSN2_RAPA	2.71298	0.84988	0.27604	51
MSN4_H2O2Hi	0.19895	0.22753	3.02776	71
MSN4_H2O2Lo	0.81629	0.15667	2.87175	19
MSN4_RAPA	2.51704	0.83717	0.09506	57
MSS11_BUT14	0.41687	0.79447	0.50271	25
Nitrogen.Depletion.1.d	1.95739	13.95895	2.53631	558
Nitrogen.Depletion.1.h	0.00000	2.54820	1.20081	350
Nitrogen.Depletion.12.h	0.19228	4.79148	2.08057	446
Nitrogen.Depletion.2.d	3.02837	12.96782	3.22712	544
Nitrogen.Depletion.2.h	0.97518	5.95842	10.78028	117
Nitrogen.Depletion.3.d	2.33525	9.32961	3.17478	653
Nitrogen.Depletion.30.min.	0.00000	8.01052	3.73559	331
Nitrogen.Depletion.4.h	0.70605	5.26004	6.35772	81
Nitrogen.Depletion.5.d	3.50530	9.93144	3.43005	635
Nitrogen.Depletion.8.h	0.00010	1.41732	1.45033	452
NRG1_H2O2Hi	0.60276	2.09861	5.45398	128
NRG1_H2O2Lo	2.60808	5.38837	0.63200	47
PHD1_BUT90	2.62629	3.20562	1.28907	113
PHO2_H2O2Hi	0.82102	0.61573	1.96545	16
PHO2_SM	0.33821	0.20126	1.60633	24
PHO4_Pi.	0.92647	0.31180	0.10323	31
PUT3_H2O2Lo	0.14073	0.73231	1.18326	90
PUT3_SM	0.28261	1.55978	2.25646	29
RCS1_H2O2Hi	0.22168	1.84747	1.79610	52
RCS1_H2O2Lo	0.07212	0.06801	0.07228	261
RCS1_SM	0.61992	0.34532	0.66510	24
RDS1_H2O2Hi	0.22580	0.25452	0.36015	46
REB1_H2O2Hi	0.02201	0.33154	0.04093	59
RIM101_H2O2Hi	0.19738	0.04228	1.66636	16
RIM101_H2O2Lo	1.75192	5.07638	0.89576	55
ROX1_H2O2Hi	1.89036	3.19913	1.03683	57
RPH1_H2O2Hi	0.14574	0.69046	0.33841	29
RPN4._H2O2Hi	0.10153	0.27755	0.09040	13
RPN4_H2O2Lo	0.00002	0.00936	0.01099	100
RTG1_RAPA	2.01830	0.93850	0.00001	31
RTG3_H2O2Hi	0.26772	0.79990	0.86302	37
RTG3_RAPA	0.30774	1.25822	3.04847	54

RTG3_SM	0.20589	1.11376	1.90816	32
SFP1_H2O2Lo	0.11598	0.83284	0.16835	18
SFP1_SM	0.00001	0.01134	0.00481	46
SKN7_H2O2Hi	0.41937	0.38331	6.01962	103
SKN7_H2O2Lo	0.67648	0.23091	9.19923	190
SOK2_BUT14	0.75164	1.50502	2.06058	73
STE12_Alpha	7.00925	1.11352	1.30628	125
STE12_BUT14	7.51951	0.27839	0.70820	135
STE12_BUT90	8.81993	0.34490	1.17518	105
steady.state.1M.sorbitol	4.94116	2.58163	0.65028	135
STP1_SM	0.96280	1.28265	1.77213	72
TEC1_Alpha	6.13045	2.41444	1.27890	43
TEC1_BUT14	2.04649	1.14808	0.14663	38
THI2_Thi.	0.62114	0.47934	2.40097	47
UME6_H2O2Hi	0.69155	1.74344	0.08478	103
X1.5.mM.diamide..10.min.	0.00019	1.81611	15.07066	152
X1.5.mM.diamide..20.min.	0.00000	4.48759	11.96224	332
X1.5.mM.diamide..30.min.	0.00002	3.93155	11.49386	293
X1.5.mM.diamide..40.min.	0.06999	3.02057	19.94591	205
X1.5.mM.diamide..5.min.	0.22488	1.14013	5.39354	30
X1.5.mM.diamide..50.min.	0.27704	5.48208	12.04464	111
X1.5.mM.diamide..60.min.	1.03293	3.71984	18.04631	123
X1.5.mM.diamide..90.min.	0.19383	1.09648	4.46554	43
X1.mM.Menadione..10.min.redo	0.67827	0.17638	2.48907	23
X1.mM.Menadione..105.min..redo	0.12507	1.28561	4.30658	54
X1.mM.Menadione..120.min.redo	0.57238	0.75913	4.88632	76
X1.mM.Menadione..160.min..redo	0.20912	0.52474	4.52153	42
X1.mM.Menadione..20.min..redo	0.64968	1.51821	6.09134	44
X1.mM.Menadione..30.min..redo	0.09164	0.29085	7.45031	48
X1.mM.Menadione..50.min.redo	1.32994	0.44845	4.00182	41
X1.mM.Menadione..80.min..redo	3.37938	2.58888	2.70606	74
X1M.sorbitol...15.min	0.00251	3.86984	5.59017	181
X1M.sorbitol...30.min	0.34124	2.15326	11.45698	71
X1M.sorbitol...45.min	1.29155	4.04069	4.30535	29
X1M.sorbitol...5.min	0.94088	0.08055	2.74586	17
X1mM.Menadione..40.min..redo	0.41977	0.51804	4.55343	50
X2.5mM.DTT.005.min.dtt.1	3.55623	4.06452	0.02679	74
X2.5mM.DTT.015.min.dtt.1	3.88271	4.64340	1.65059	37
X2.5mM.DTT.030.min.dtt.1	0.45326	0.71588	1.74154	46
X2.5mM.DTT.045.min.dtt.1	0.12826	0.01097	2.76095	48
X2.5mM.DTT.060.min.dtt.1	0.34281	0.29323	2.55470	67
X2.5mM.DTT.090.min.dtt.1	0.22055	0.11739	3.36298	67
X2.5mM.DTT.120.min.dtt.1	0.04277	1.05782	5.21898	160
X2.5mM.DTT.180.min.dtt.1	0.00153	0.51894	2.01835	207
X29C..1M.sorbitol.to.33C....NO.sorbitol...15.minutes	1.39328	2.34256	3.17575	49
X29C..1M.sorbitol.to.33C....NO.sorbitol...30.minutes	1.82630	1.58167	3.76089	38

X29C..1M.sorbitol.to.33C....NO.sorbitol...5.minutes	2.22719	0.94294	5.27846	82
X29C..1M.sorbitol.to.33C...1M.sorbitol...15.minutes	2.04625	1.23420	12.79633	97
X29C..1M.sorbitol.to.33C...1M.sorbitol...5.minutes	1.07816	1.29272	9.15032	69
X29C.to.33C...15.minutes	1.85113	2.00499	14.39222	94
X29C.to.33C...30.minutes	0.85385	0.80664	1.71251	102
X29C.to.33C...5.minutes	2.63402	2.58856	9.04835	226
X33C.vs..30C...90.minutes	0.27265	0.77126	0.21950	22
X37C.to.25C.shock...15.min	9.68086	2.39389	3.10558	267
X37C.to.25C.shock...30.min	2.26742	1.34081	2.76777	142
X37C.to.25C.shock...45.min	2.60182	2.41667	7.10059	142
X37C.to.25C.shock...60.min	3.73924	1.07555	6.40255	150
X37C.to.25C.shock...90.min	0.29948	0.94947	4.59196	57
XBP1_H2O2Lo	4.79943	3.58533	0.40665	77
YAP1_H2O2Lo	0.00746	0.82772	0.88762	38
YAP6_H2O2Hi	2.57334	4.21857	0.62519	90
YAP6_H2O2Lo	1.00109	1.87761	1.43104	60
YAP7_H2O2Hi	0.03334	0.41578	1.79512	140
YAP7_H2O2Lo	0.05524	0.57267	2.65722	151
