# Supplementary material for: Stochastic and Regulatory Role of Chromatin Silencing in Genomic Response to Environmental Changes
Source: PLoS One. 2008 Aug 20;3(8):e3002. doi: 10.1371/journal.pone.0003002 (PMC2500160; doi:10.1371/journal.pone.0003002)
Supplement: Table S7 — Analysis of Gene Ontology categories. Genes in each category were compared with the rest of genes and its significance was reported as -log10 (P value). The table contains Gene Ontology categories, the silencing activity of genes in each category (Sir2/3/4 and Set1), the sum of the two silencing scores (Silencing), and the noise of genes in each set as measured in rich medium (Noise (ypd)). Selected categories are shown in the same color-code as the rectangles and circles in Fig. 4D. (0.01 MB PDF) [file pone.0003002.s013.pdf]

Table S7

Gene Ontology	Sir2/3/4	Set1	Silencing	Noise (ypd)
**meiosis**	5.15246	2.73747	7.88993	0.02181
**meiotic cell cycle**	5.15246	2.73747	7.88993	0.02181
**M phase of meiotic cell cycle**	5.15246	2.73747	7.88993	0.02181
**plasma membrane**	3.44780	3.40590	6.85370	3.48456
**reproductive sporulation**	0.88829	5.94299	6.83128	0.12640
**sporulation (sensu Fungi)**	0.88829	5.94299	6.83128	0.12640
**sporulation**	1.53524	5.24434	6.77958	0.09587
**response to pheromone**	5.60563	1.05962	6.66525	0.13674
**external encapsulating structure**	5.64737	1.01539	6.66277	3.49671
**chitin- and beta-glucan-containing cell wall**	5.64737	1.01539	6.66277	3.49671
**cell wall**	5.64737	1.01539	6.66277	3.49671
**reproduction**	3.42227	3.00400	6.42627	0.04275
**cellular developmental process**	1.87794	4.20617	6.08411	0.06360
**cell differentiation**	1.87794	4.20617	6.08411	0.06360
DNA-directed DNA polymerase activity	5.39156	0.59300	5.98456	0.02915
rRNA processing	0.00030	5.90716	5.90746	0.52297
ribosome biogenesis and assembly	0.00003	5.70717	5.70721	1.00876
meiosis I	3.93431	1.65683	5.59114	0.13173
rRNA metabolic process	0.00043	5.51490	5.51532	0.57430
**oxidoreductase activity, acting on CH-OH group of donors**	0.46357	4.95574	5.41930	9.88909
ribonucleoprotein complex biogenesis and assembly	0.00004	5.32963	5.32967	0.50401
meiotic recombination	3.70292	1.61086	5.31377	0.26028
sexual reproduction	4.01411	1.04259	5.05670	0.75636
conjugation	4.01411	1.04259	5.05670	0.75636
conjugation with cellular fusion	4.01411	1.04259	5.05670	0.75636
**oxidoreductase activity, acting on the CH-OH group of donors, NAD or NADP as accepto**	0.72268	4.32311	5.04580	8.94814
reproductive process	1.60601	3.16778	4.77379	0.01708
vitamin biosynthetic process	0.62060	4.04467	4.66528	0.82628

water-soluble vitamin biosynthetic process	0.62060	4.04467	4.66528	0.82628
response to pheromone during conjugation with cellular fusion	3.25157	1.38861	4.64017	0.33234
regulation of conjugation with cellular fusion	3.80317	0.81454	4.61771	0.31494
signal transduction during conjugation with cellular fusion	3.80317	0.81454	4.61771	0.31494
pheromone-dependent signal transduction during conjugation with cellular fusion	3.80317	0.81454	4.61771	0.31494
regulation of conjugation	3.80317	0.81454	4.61771	0.31494
vitamin metabolic process	0.26644	4.29934	4.56578	3.80698
water-soluble vitamin metabolic process	0.26644	4.29934	4.56578	3.80698
G-protein coupled receptor protein signaling pathway	2.56480	1.87018	4.43499	0.19351
reproductive process in single-celled organism	0.88600	3.54598	4.43197	0.02741
reproductive cellular process	0.88600	3.54598	4.43197	0.02741
nucleolus	0.00256	4.06201	4.06457	0.62628
secondary active transmembrane transporter activity	2.19286	1.82492	4.01778	2.20209
