# Supplementary material for: Stochastic and Regulatory Role of Chromatin Silencing in Genomic Response to Environmental Changes
Source: PLoS One. 2008 Aug 20;3(8):e3002. doi: 10.1371/journal.pone.0003002 (PMC2500160; doi:10.1371/journal.pone.0003002)
Supplement: Table S8 — Stress response of oxidoreductase genes. We analyzed genes belonging to the two categories identified as ‘oxidoreductase activity’ in Table S7. The table reports stress conditions as defined from the expression profiles and transcription-factor location analyses, and the responsiveness of the genes to each stress condition, which is represented as -log10 (P value). Shown in red are stress conditions where the responsiveness score is greater than the threshold. (0.02 MB PDF) [file pone.0003002.s014.pdf]

Table S8

stress condition	responsiveness
**constant.0.32.mM.H2O2..50.min..redo**	10.16967
**X1.5.mM.diamide..50.min.**	10.12476
**X1.5.mM.diamide..30.min.**	9.60098
**MAL33_H2O2Hi**	9.51034
**X1.5.mM.diamide..40.min.**	9.46265
**X1.mM.Menadione..50.min.redo**	9.10940
**PHO2_H2O2Hi**	8.34421
**X1.5.mM.diamide..20.min.**	8.19445
**constant.0.32.mM.H2O2..60.min..redo**	8.01224
**RDS1_H2O2Hi**	7.86457
**X1.5.mM.diamide..60.min.**	7.67790
**aa.starv.2.h**	7.50441
**X1.5.mM.diamide..10.min.**	7.12504
**aa.starv.1.h**	6.95960
**constant.0.32.mM.H2O2..80.min..redo**	6.17336
**GZF3_RAPA**	6.10360
**X1mM.Menadione..40.min..redo**	6.08165
X1.5.mM.diamide..90.min.	5.35812
constant.0.32.mM.H2O2..100.min..redo	5.19361
aa.starv.6.h	5.16202
Nitrogen.Depletion.2.h	4.86709
RPH1_H2O2Hi	4.82796
X1.mM.Menadione..80.min..redo	4.77925
X1.mM.Menadione..105.min..redo	4.74282
diauxic.shift.timecourse.18.5.h	4.72623
X1.mM.Menadione..30.min..redo	4.71487
heat.shock.33.to.37..20.minutes	4.56492
XBP1_H2O2Lo	4.51483
constant.0.32.mM.H2O2..120.min..redo	4.35662
RTG3_RAPA	4.34976
RIM101_H2O2Hi	4.13256
SIG1_H2O2Hi	4.13256
IME4_H2O2Hi	4.12747
X37C.to.25C.shock...45.min	4.10700
ROX1_H2O2Hi	4.10196
YAP7_H2O2Lo	3.93031
BAS1_SM	3.91415
YAP6_H2O2Hi	3.76100
YAP1_H2O2Lo	3.68863
GAL4_RAFF	3.59181
aa.starv.0.5.h	3.53335
NRG1_H2O2Hi	3.52158
ARR1_H2O2Hi	3.50529
YAP5_H2O2Hi	3.50529
diauxic.shift.timecourse.20.5.h	3.37940
constant.0.32.mM.H2O2..30.min..redo	3.30101

Nitrogen.Depletion.2.d	3.27345
aa.starv.4.h	3.13906
YAP7_H2O2Hi	3.12924
Nitrogen.Depletion.1.d	3.12825
SOK2_BUT14	3.10540
Nitrogen.Depletion.3.d	3.07903
Nitrogen.Depletion.5.d	3.05690
constant.0.32.mM.H2O2..160.min..redo	2.96587
X1.mM.Menadione..160.min..redo	2.89254
Nitrogen.Depletion.4.h	2.87166
PHO2_SM	2.84705
MSN2_H2O2Hi	2.83060
diauxic.shift.timecourse.13.5.h	2.80054
MSS11_BUT14	2.73877
IME1_H2O2Hi	2.70652
HSF1_HEAT	2.70652
X1.5.mM.diamide..5.min.	2.67464
RCS1_H2O2Hi	2.64989
Nitrogen.Depletion.12.h	2.63836
heat.shock.17.to.37..20.minutes	2.61756
dtt.060.min.dtt.2	2.60443
constant.0.32.mM.H2O2..20.min..redo	2.60438
constant.0.32.mM.H2O2..10.min..redo	2.60178
heat.shock.21.to.37..20.minutes	2.59306
X29C..1M.sorbitol.to.33C....NO.sorbitol...15.minutes	2.51554
Nitrogen.Depletion.8.h	2.33235
Heat.Shock.15.minutes.hs.1	2.29054
X1.mM.Menadione..10.min.redo	2.28391
heat.shock.25.to.37..20.minutes	2.27155
Heat.Shock.30.minutes.hs.1	2.24009
AFT2_H2O2Hi	2.21956
heat.shock.29.to.37..20.minutes	2.18641
UGA3_RAPA	2.15487
GCN4_SM	2.07427
RTG3_H2O2Lo	2.03737
GLN3_RAPA	1.99353
Heat.Shock.015.minutes..hs.2	1.99112
Nitrogen.Depletion.1.h	1.97187
X1M.sorbitol...15.min	1.91130
dtt.240.min.dtt.2	1.87830
STP1_SM	1.83256
X37C.to.25C.shock...15.min	1.82434
Nitrogen.Depletion.30.min.	1.79039
Heat.Shock.000.minutes..hs.2	1.76679
UGA3_SM	1.75624
MOT3_H2O2Hi	1.75624
Hypo.osmotic.shock...60.min	1.75265
GCN4_RAPA	1.74498
X29C..1M.sorbitol.to.33C...1M.sorbitol...15.minutes	1.74333

constant.0.32.mM.H2O2..40.min..rescan	1.66702
GZF3_H2O2Hi	1.64429
RPN4._H2O2Hi	1.64429
Heat.Shock.20.minutes.hs.1	1.57349
X29C..1M.sorbitol.to.33C...1M.sorbitol...30.minutes	1.52749
Hypo.osmotic.shock...30.min	1.52141
Heat.Shock.000.minutes..hs.2.1	1.51755
dtt.000.min..dtt.2	1.48575
Heat.Shock.000.minutes.hs.2	1.46001
MSN2_H2O2Lo	1.45276
NRG1_H2O2Lo	1.45276
X29C.to.33C...15.minutes	1.40276
dtt.015.min.dtt.2	1.39944
Hypo.osmotic.shock...5.min	1.39760
PUT3_H2O2Lo	1.39571
X2.5mM.DTT.015.min.dtt.1	1.38552
MOT3_SM	1.38331
X29C..1M.sorbitol.to.33C....NO.sorbitol...5.minutes	1.33838
Heat.Shock.030inutes..hs.2	1.32052
Heat.Shock.10.minutes.hs.1	1.30336
X29C.to.33C...5.minutes	1.30205
X1M.sorbitol...30.min	1.29317
DAL81_RAPA	1.28536
X1.mM.Menadione..20.min..redo	1.28429
Heat.Shock.60.minutes.hs.1	1.24269
RPN4_H2O2Lo	1.21897
Heat.Shock.060.minutes..hs.2	1.20712
GCR2_SM	1.20122
MSN4_H2O2Lo	1.19617
X1M.sorbitol...60.min	1.19054
MSN4_RAPA	1.17786
dtt.030.min..dtt.2	1.17384
Heat.Shock.40.minutes.hs.1	1.15806
X37C.to.25C.shock...30.min	1.14278
X1M.sorbitol...5.min	1.12450
dtt.120.min.dtt.2	1.12004
SIP4_SM	1.09788
X29C.to.33C...30.minutes	1.02393
AFT2_H2O2Lo	1.01048
Hypo.osmotic.shock...45.min	0.98783
MSN4_H2O2Hi	0.91611
Heat.Shock.05.minutes.hs.1	0.89253
LEU3_SM	0.88152
GAT1_RAPA	0.88152
diauxic.shift.timecourse.15.5.h	0.88115
X37C.to.25C.shock...90.min	0.86294
MET4_SM	0.85348
X29C..1M.sorbitol.to.33C...1M.sorbitol...5.minutes	0.83376
X2.5mM.DTT.005.min.dtt.1	0.81001

X2.5mM.DTT.180.min.dtt.1	0.79255
X1M.sorbitol...120.min	0.78037
FKH2_H2O2Lo	0.77895
X2.5mM.DTT.090.min.dtt.1	0.76949
RTG3_SM	0.75684
steady.state.1M.sorbitol	0.75645
X1M.sorbitol...45.min	0.74726
X33C.vs..30C...90.minutes	0.72393
CBF1_SM	0.67454
X2.5mM.DTT.060.min.dtt.1	0.66900
TEC1_BUT14	0.64590
DAL80_RAPA	0.61545
GAL4_GAL	0.60121
X2.5mM.DTT.030.min.dtt.1	0.57620
X2.5mM.DTT.120.min.dtt.1	0.55207
X1M.sorbitol...90.min	0.54964
X1.mM.Menadione..120.min.redo	0.53133
GAT1_SM	0.52697
THI2_Thi.	0.52697
Heat.Shock.80.minutes.hs.1	0.49701
MSN2_RAPA	0.48605
ASH1_BUT14	0.48605
Hypo.osmotic.shock...15.min	0.45177
RIM101_H2O2Lo	0.45037
DAL82_SM	0.41895
YAP6_H2O2Lo	0.41167
MBP1_H2O2Lo	0.40460
Diauxic.Shift.Timecourse...0.h	0.33497
dtt.480.min.dtt.2	0.33119
DAL81_SM	0.26351
HAP4_SM	0.26351
PHO2_H2O2Lo	0.26351
RPH1_H2O2Lo	0.26351
PHO2_Pi.	0.26351
diauxic.shift.timecourse11.5	0.26285
KSS1_BUT90	0.24928
MTH1_GAL	0.24928
X29C..1M.sorbitol.to.33C....NO.sorbitol...30.minutes	0.24570
CAD1_H2O2Hi	0.23000
KSS1_Alpha	0.23000
SKN7_H2O2Lo	0.22322
ADR1_SM	0.22252
SFP1_H2O2Hi	0.22252
MOT3_H2O2Lo	0.22252
UME6_H2O2Hi	0.22106
RTG1_SM	0.21591
MIG2_H2O2Hi	0.21591
ROX1_H2O2Lo	0.21591
MSN4_Acid	0.21591

YAP1_H2O2Hi	0.20995
X2.5mM.DTT.045.min.dtt.1	0.20680
PDR1_H2O2Lo	0.20450
diauxic.shift.timecourse.9.5.h	0.20336
PHD1_BUT90	0.19582
ARG80_SM	0.19481
YJL206C_H2O2Lo	0.19481
SKN7_HEAT	0.18633
YJL206C_H2O2Hi	0.18244
MET31_SM	0.17876
RPH1_SM	0.17876
ADR1_HEAT	0.17876
HSF1_H2O2Hi	0.17049
MAC1_H2O2Hi	0.16566
SFP1_H2O2Lo	0.16566
MBP1_H2O2Hi	0.15263
RCS1_SM	0.14956
HAP4_H2O2Lo	0.14956
CAD1_SM	0.14488
ARG81_SM	0.14048
PUT3_SM	0.13838
RTG1_RAPA	0.13434
MSN2_Acid	0.13434
PHO4_Pi.	0.13434
ARO80_SM	0.13240
CHA4_SM	0.12341
HAP5_SM	0.12010
X37C.to.25C.shock...60.min	0.11963
MET32_SM	0.11694
HAP2_RAPA	0.11541
DIG1_BUT90	0.11541
TEC1_Alpha	0.11392
SFP1_SM	0.10961
GLN3_SM	0.10426
DAL82_RAPA	0.09696
REB1_H2O2Hi	0.09360
DIG1_BUT14	0.08541
DIG1_Alpha	0.07736
CIN5_H2O2Hi	0.07333
HSF1_H2O2Lo	0.05911
SKN7_H2O2Hi	0.05853
STE12_BUT90	0.05738
MCM1_Alpha	0.05626
FKH2_H2O2Hi	0.05571
STE12_Alpha	0.04729
RCS1_H2O2Lo	0.04596
CIN5_H2O2Lo	0.04553
STE12_BUT14	0.04305
Heat.Shock.005.minutes..hs.2	0.03696

FHL1_RAPA	0.02602
FHL1_SM	0.02324
MET28_SM	0.00000
RAP1_SM	0.00000
FHL1_H2O2Hi	0.00000
YAP3_H2O2Hi	0.00000
MAL33_H2O2Lo	0.00000
REB1_H2O2Lo	0.00000
PHD1_BUT14	0.00000
RLM1_BUT14	0.00000
MIG1_GAL	0.00000
RGT1_GAL	0.00000
GAT1_HEAT	0.00000
MSN2_HEAT	0.00000
YAP1_HEAT	0.00000
